# Validation of an FBA model for *Pichia pastoris* in chemostat cultures

**DOI:** 10.1186/s12918-014-0142-y

**Published:** 2014-12-24

**Authors:** Yeimy Morales, Marta Tortajada, Jesús Picó, Josep Vehí, Francisco Llaneras

**Affiliations:** MICElab, IIIA, Universitat de Girona, Campus Montilivi, P4, Girona, 17071 Spain; BIÓPOLIS S.L., C/Catedrático Agustín Escardino Benlloch, 9, 46980 Paterna, Valencia Spain; Institut Universitari d Automàtica i Informàtica Industrial, Universitat Politècnica de València, Camino de Vera s/n, Edificio 5C, 46022 Valencia, Spain

**Keywords:** Constraint- based model, Flux balance analysis, Possibilistic metabolic flux analysis, *Pichia pastoris*

## Abstract

**Background:**

Constraint-based metabolic models and *flux balance analysis (FBA)* have been extensively used in the last years to investigate the behavior of cells and also as basis for different industrial applications. In this context, this work provides a validation of a small-sized FBA model of the yeast *Pichia pastoris.* Our main objective is testing how accurate is the hypothesis of maximum growth to predict the behavior of *P. pastoris* in a range of experimental environments.

**Results:**

A constraint-based model of P. pastoris was previously validated using metabolic flux analysis (MFA). In this paper we have verified the model ability to predict the cells behavior in different conditions without introducing measurements, experimental parameters, or any additional constraint, just by assuming that cells will make the best use of the available resources to maximize its growth. In particular, we have tested FBA model ability to: (a) predict growth yields over single substrates (glucose, glycerol, and methanol); (b) predict growth rate, substrate uptakes, respiration rates, and by-product formation in scenarios where different substrates are available (glucose, glycerol, methanol, or mixes of methanol and glycerol); (c) predict the different behaviors of P. pastoris cultures in aerobic and hypoxic conditions for each single substrate. In every case, experimental data from literature are used as validation.

**Conclusions:**

We conclude that our predictions based on growth maximisation are reasonably accurate, but still far from perfect. The deviations are significant in scenarios where *P. pastoris* grows on methanol, suggesting that the hypothesis of maximum growth could be not dominating in these situations. However, predictions are much better when glycerol or glucose are used as substrates. In these scenarios, even if our FBA model is small and imposes a strong assumption regarding how cells will regulate their metabolic fluxes, it provides reasonably good predictions in terms of growth, substrate preference, product formation, and respiration rates.

**Electronic supplementary material:**

The online version of this article (doi:10.1186/s12918-014-0142-y) contains supplementary material, which is available to authorized users.

## Background

*Pichia pastoris* is a methylotrophic yeast widely recognized as a suitable expression system for basic research and industrial application [1]. More than 500 proteins have been expressed using this system due to (a) the possibility to grow cultures to very high cell densities. (b) The existence of methanol-inducible alcohol oxidase promoters (AOX). (c) its ability to produce post-translational modifications, and (d) the good protein yield/cost ratio.

As any other living cell*, P. pastoris* cells are complex systems, but they can be represented as an array of reactions that convert raw materials into energy and building blocks. These collections of chemical reactions form a metabolic network; and these metabolic networks can be encoded in an ***mxn*** matrix, with ***m*** metabolites and ***n*** reactions, called stoichiometric matrix [2-4]. From these networks, a constraint-based model can be derived by imposing a mass balance around the metabolites assumed to be balanced —mostly internal ones—, and by constraining those reactions that are assumed to be irreversible. This way, a constraint-based model defines a space of feasible flux distributions, *i.e*., a space of all the metabolic behaviors that the cells can show in different conditions [5,6]. These models have the advantage of not requiring knowledge about kinetic parameters, which are rarely known for most intracellular reactions.

The space of feasible flux distribution can be still reduced by adding more constraints, such as context-dependent assumptions. As a result, there are several methodologies employed with different purposes and making use of different mathematical frameworks, but they all have in common the use of a constraint-based modeling approach [5].Two popular approaches are metabolic flux analysis (MFA) and flux balance analysis (FBA). MFA combines the constraint-based model with a set of experimental measurements, usually of extracellular fluxes, to perform estimations [7]. FBA also uses a constraint-based model, but it incorporates an assumption of optimal cell behavior [2,8-10].

In particular, FBA is a framework to get predictions from a constraint-based model using optimization [2,6,8,11]. FBA predictions are based on assuming that cells, due to evolutionary pressure, have evolved to be optimal in a particular (and known) way. This approach reduces the space of feasible flux distributions generated by the constraint-based model by incorporating «input» constraints —typically bounds for the uptake fluxes, based on known capacities or the availability of substrates—, and defining an objective function based on an assumption of optimal cell behavior. Often, the objective function chosen is the maximization of the biomass growth rate [12,13]. However, many other objective functions have been proposed, such as the maximization of ATP production rate [14] or the minimization of total flux [15].

Even if FBA predictions based on the hypothesis of maximal growth rate have been shown to be reasonably accurate in several studies, their limitations have been also investigated [16]. It has been argued that the assumption is well justified in many cases, but not in all situations [10]. Similar conclusions were drawn by Shuetz et al., when the authors performed a systematic evaluation of different objective functions in order to predict intracellular fluxes of *E. coli* cultures by invoking optimality principles [13]. They found that no single objective function was able to accurately predict the behavior that cells shown in all the conditions. These limitations are the basis to investigate more sophisticated objective functions and also for dealing with multiple criteria simultaneously, by means of Pareto surface and other analytical tools [17,18].

In this paper, we present the validation of a FBA (constraint-based) model of *P. pastoris* based on a small-sized metabolic network. In line with previous works done with small models of other organisms, such as *E. coli* [19,20], *S. cerevisiae* [21,22] or *Aspergillus niger* [23], with a less studied organism as *P. pastoris*. Our main objective is testing how accurate is the hypothesis of maximum growth rate to predict the cells behavior in a range of experimental environments. The underlying constraint-based model of *P. pastoris* was previously validated against experimental data using MFA [24]. Now we will test the FBA model ability to give reasonable predictions without incorporating measurements, just by assuming that cells will make the best use of the available resources.

## Methods

### Constraint based metabolic model

Along this paper, a constraint-based model of *P. pastoris* has been used. The model is a modified version of the one previously described and validated in [24,25]. It is a standard constraint-based model, as those described in [5] or [2]. The model was derived from a set of central metabolic reactions. These reactions are then translated into constraints by assuming that intracellular metabolites are at steady-state (and disregarding the dilution effect). Then, another set of inequality constraints is incorporated by imposing irreversibility to some reactions. This procedure results in a set of model constraints (MOC) that defines a space of feasible steady state flux distributions, as follows:

1 $$ M\mathcal{O}C=\left\{\begin{array}{c}\hfill \boldsymbol{N}\cdot \mathbf{v}=\mathbf{0}\hfill \\ {}\hfill \boldsymbol{D}\cdot \mathbf{v}\ge \mathbf{0}\hfill \end{array}\right. $$

Where **N** is a stoichiometric matrix, with *m* metabolites and *n* reactions, the vector **v** is the vector of reaction fluxes, which represent the mass flow through each of the *n* reactions in the network. The matrix **D**, is a diagonal matrix with **D**_ij_ = 1 if the flux is irreversible and null otherwise.

### Consistency analysis of experimental data

To validate our model predictions, several experimental datasets corresponding to different *P. pastoris* chemostat experiments have been collected from literature. Each dataset contains experimental measurements of several extracellular fluxes (*e.g.,* biomass growth, glucose uptake rate, oxygen uptake rate, *etc*.). However, these experiments came from different sources, correspond to cultures of different strains, and have been obtained following different experimental protocols. For this reason the consistency of each dataset has been evaluated beforehand using two different methods: (a) a simple carbon balance, and (b) a possibilistic consistency analysis against our stoichiometric model.

### Carbon balance

The consistency of each experimental dataset has been evaluated checking that the measurements fulfilled a C-mol balance. This test could only be performed when measurements for the main uptake and production fluxes of carbon sources were available, which generally means that all substrates (glucose, glycerol and methanol), biomass and CO_2_ rates were measured, as well as the main possible byproducts (ethanol, pyruvate, and citrate). The actual elemental composition of biomass and ash content were taken into account whenever available; otherwise a mean composition was used. A general elemental composition for recombinant protein was taken from [3]. In those cases where heterologous protein was measured, it was included in the carbon balance; however, as the carbon content was small, it was neglected in those datasets where protein production was unknown.

In summary, for 52 datasets the carbon balance was checked based on measurements of glucose, glycerol, methanol, CO_2_, biomass, protein, pyruvate, ethanol, and citrate (note: in some cases the byproducts were not measured, but reported negligible). For datasets 17, 18 and 50–52 protein production was unknown, but its carbon content was assumed to be negligible. Finally, datasets 29 to 45 and 53 to 55 could not be checked because the CO_2_ production rate was unknown.

### Possibilistic MFA

As a complementary test, and also to deal with those experimental datasets lacking a carbon-balance, we perform a different consistency analysis based on Possibilistic MFA. The method was described in [5,26] and applied in [24,25]. Details can be found in those works, but a short description follows. First, we describe the Possibilistic MFA method, and then we explain how it can be used to perform a consistency analysis.

Possibilistic MFA takes into account that experimental measurements are imprecise and do not exactly satisfy the constraints in (1). All measurements are thus considered relatively uncertain, as follows: **w**_**m**_ = **v**_**m**_ + **e**_**m**_, where **e**_**m**_ is a vector containing the errors (or deviations) between the actual fluxes **and** their measured values. Similarly, these measurement errors can be represented with two sets of non-negative variables, ε and μ:

2 $$ M\upvarepsilon C=\left\{\begin{array}{c}\hfill {\mathbf{w}}_{\mathbf{m}}={\mathbf{v}}_{\mathbf{m}}+{\upvarepsilon}_1-{\upmu}_1+{\upvarepsilon}_2-{\upmu}_2\hfill \\ {}\hfill {\upvarepsilon}_1,{\upmu}_1\ge 0\hfill \\ {}\hfill 0\le {\upvarepsilon}_2\le {\upvarepsilon}_2^{\max}\hfill \\ {}\hfill 0\le {\upmu}_2\le {\upmu}_2^{\max}\hfill \end{array}\right. $$

Each candidate solution of (1) and (2) can be denoted as δ. Then, we (as users) define a function that assigns possibility in [0, 1] to each solution, ranging between impossible and fully possible. A simple way is using a linear cost index as:

3 $$ \mathbf{J}\left(\updelta \right)=\upalpha \bullet {\varepsilon}_1+\beta \bullet {\mu}_1 $$

Then, the possibility of each solution can be defined as:

4 $$ \uppi\ \left(\updelta \right)= \exp \Big(-\mathbf{J}\left(\updelta \right)\kern2em \delta \subset \Delta $$

Where α y β are row vectors of user defined, sensor accuracy coefficients. The results can be interpreted as “**v**_**m**_ = **w** is fully possible; the more **v**_**m**_ and **w** differ, the less possible such situation is”. In particular, and for all our computations, the bounds ε_2_^max^ and μ_2_^max^ have been chosen to define an interval of fully possible values around the measured ones (±5% deviation); while the weights α and β have been chosen to a decreasing possibility to larger deviations (e.g., deviations larger than ±20% have a possibility of lower than π = 0.1). More details can be found in [25].

At this point, Possibilistic MFA provides flux estimates accounting for uncertainty. For instance, the simplest flux estimate **v**_mp_ in δ_mp_ is given by a maximum possibility (minimum cost) solution of the constraint satisfaction problem (1)-(2), which can be obtained solving a linear programming (LP) problem.

5 $$ \begin{array}{cc}\hfill {\mathbf{J}}^{\mathbf{min}}= \min {}_{\varepsilon, \mu, \nu}\mathbf{J}\hfill & \hfill s.t\ \left\{M\mathcal{O}C\cap M\upvarepsilon C\right.\hfill \end{array} $$

This most possible solution given by (5) has an associated degree of possibility:

6 $$ {\pi}^{mp}= \exp \left(-{J}^{min}\right) $$

This value in [0, 1] provides our consistency check. This value π^mp^ is the possibility of the most possible flux distribution. It is grading the degree of consistency between different measurements, and between the measurements (2) and the model constraints in (1). A possibility equal to one must be interpreted as a complete consistency, while lower values imply that there is some error in measurements or in the model.

Finally, there is a similar way of express the degree of consistency provided by the possibilistic method. In this case, we calculate the percentage of measurements error (in ε_2_^max^, μ_2_^max^) that must be allowed to find a solution with possibility equal to 1. We denote this degree of “assumed error” as AE index. Clearly, the larger this index is, the more inconsistent measurements are. For example, an AE index of 10% implies that a 10% of flexibility is required around all the measurements to find a solution that fulfills simultaneously the measurements and model constraints.

Note: This consistency analysis assumes that model constraints are accurate; but let us remark that the FBA hypothesis, which will be evaluated along this paper, has not been included so far. The model used in the consistency analysis was validated before and has been proved to be relatively reliable [24,25].

### Flux balance analysis

Several flux balance analysis (FBA) simulations have been performed. As stated in the backgrounds section, FBA is a methodology to get predictions from a constraint-based model by assuming that the cells behave optimally. In this way, predictions are obtained by solving an optimization problem: maximize the (hypothetical) cells objective function subject to the constraints that are imposed by the model.

If the objective function is linear and the constraints are linear equalities and inequalities —which is the case for all our computations—, the FBA problem can be formulated as a linear programming problem. In this case, predictions can be obtained following a simple and efficient four-step procedure.

First: define a set of model constraints (MOC), such as in (1). These constraints are always the same for a given organism, independently of its environment and particular circumstances.

Second: incorporate context-dependent constraints, which represent the scenario that the modeled organism is facing in a particular case. For example, these constraints define which substrates are available or if there is oxygen in the media. In general, these constraints will be inequalities:

7 $$ {\mathbf{v}}_u^{min}\ge {\mathbf{v}}_u\ge {\mathbf{v}}_u^{max} $$

Third: define a biologically relevant objective function Z that is assumed to represent the cells objective, as result of evolutionary pressure. In all our computations this objective will be to maximize growth. The objective function is defined as follows (where d is column vector of size *n* with zeros in every position but the one corresponding to the biomass growth):

8 $$ Z=\mathbf{d}\cdot \mathbf{v} $$

Fourth: finally, predictions are obtained by solving a linear programing problem to compute the flux distribution that makes the optimal use of the available resources, (*i.e.*, that maximizes the objective function Z).

9 $$ {\mathbf{v}}^{opt}= \max {}_{\mathbf{v}}Z\ s.t\ \left\{\begin{array}{c}\hfill \boldsymbol{N}\cdot \mathbf{v}=0\kern0.5em \hfill \\ {}\hfill\ \boldsymbol{D}\cdot \mathbf{v}\ge 0\hfill \\ {}\hfill {\mathbf{v}}_u^{min}\ge {\mathbf{v}}_u\ge {\mathbf{v}}_u^{max}\ \hfill \end{array}\right\} $$

All FBA computations have been performed with MATLAB (MathWorks Inc., 2009) and YALMIP Toolbox [27].

## Results and discussion

### *P. pastoris* constraint-based model building

**Figure 1:**
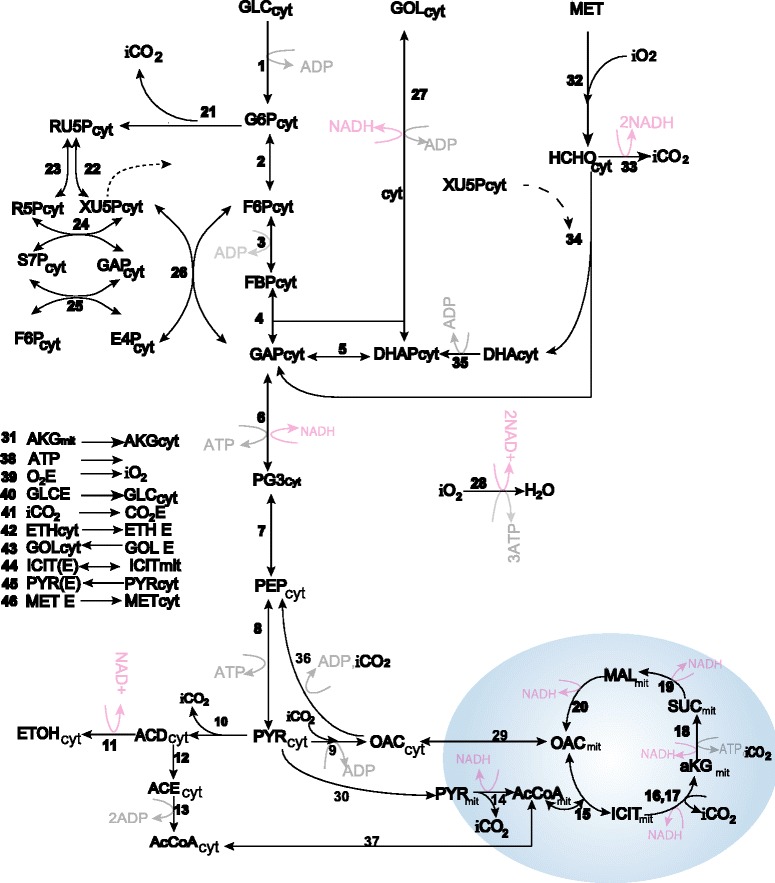
**Metabolic network of** ***P. pastoris.*** Metabolic network for the *Pichia pastoris* model. For the sake of clarity, the reactions representing biomass growth and ATP balance have not been included in the scheme (they can be found in the Additional file 1).

Along this paper, a small-sized, constraint-based model of *P. pastoris* shown in Figure 1 will be used. The model is a modified version of the one previously described and validated in [24], which was based in a previous model by Dragosits *et al.* [28] it is a standard constraint-based model, whose generalities are described in [5] or [2].

As a constraint based model, it was derived from the knowledge about *P. pastoris* metabolic network. The model is not a comprehensive representation of *P. pastoris* metabolism, but it includes the main catabolic pathways (Embden-Meyerhoff-Parnas pathway, citric acid cycle, pentose phosphate and fermentative pathways), considers the uptake of several carbon sources (glucose, glycerol, and methanol) and accounts for biomass growth and ATP balance. Metabolites such as NAD, AcCoA, oxaloacetate, or pyruvate are considered in both cytosolic and mitochondrial pools.

Two new reactions have been incorporated to the model described in [24] in the pyruvate metabolism and in the mitochondrial transport. The new reactions are:

Reaction 36: *ATP* + *Oxaloacetate* → *ADP* + *Phosphoenolpyruvate* + *CO*2.

Reaction 37: *Acetyl* − *CoAmit* ↔ *Acetyl* − *CoA*.

The model contains 47 metabolites and 48 metabolic reactions. There are 37 internal metabolites that are assumed balanced, which define a 37x48 stoichiometric matrix **N** with 11 degrees of freedom. All internal reactions are considered irreversible, except for reactions; 2–8, 15, 22–27, 29, 34, 37 and 44. The matrix and the list of reactions are given in the Additional file 1.

### *P. pastoris* FBA models

Along this paper the word “model” is used to denote two different representations of *P. pastoris.* The first one is the constraint-based model of *P. pastoris* that we have already defined which contains only information regarding its central metabolism and reactions irreversibilities. The second type of model emerges when we combine this constraint-based model with a biological objective for the cells (maximizing growth), so that we obtain a complete FBA model as defined in the methods sections. Please recall that the main goal of this paper is to evaluate the validity of the second model, i.e., the validity of assuming that *P. pastoris* cells objective is maximizing its growth rate. Hereinafter, we will denote this second model as *FBA model.*

### Recompilation and analysis of experimental data

Thus, the main goal of this paper is to validate the predictions of an FBA model. To do that, experimental datasets from different chemostat experiments have been collected from literature. We collected data from 72 chemostat experiments that correspond to *P. pastoris* cultures growing on methanol, glycerol, glucose or mixtures of these substrates. Each dataset is defined by a set of experimental measurements of several extracellular fluxes (*e.g.,* biomass growth, glucose uptake rate, oxygen uptake rate, *etc*.). The number of available measurements in each dataset is not always the same, mostly because gas measurements are sometimes unavailable. Most datasets correspond to recombinant strains, resulting in the production of a heterologous protein. All datasets can be found in Additional file 2.

Please notice that the experimental datasets come from different sources and correspond to experiments with different strains and different experimental protocols. For this reason, before using them, the consistency of each dataset has been evaluated using two different methods: (a) a simple carbon-balance, and (b) a possibilistic consistency analysis against our stoichiometric model. Both methods are described in detail in the methods section. The complete results of these analyses can be found in the Additional file 2.

The carbon-balance test of consistency could only be performed with 52 datasets for which CO_2_ measurements were available. The consistency is reasonably good for the majority of the tested datasets, with a deviation minor than 10% in carbon content for datasets; 1–4, 7–14, 46–48, 50, 51, 56–72. Only a few datasets (5, 6, 15, 24–28, 49) have a deviation higher than 10%.

To provide further validation of the data, and deal with those datasets which consistency cannot be evaluated with a carbon balance, a possibilistic MFA consistency test was also applied. Again, most of the datasets are highly consistent with the model: 72% are fully possible and only 4 in 72 datasets have an AE index larger than 15% —this includes the intrinsic uncertainty of any measure (*e.g.* calibration errors, offsets, etc.).

As a result of the analysis, datasets 5, 6, and 15 have been classified as inconsistent with both methods. This result suggests that measurement errors are likely in those datasets. We have decided to keep all datasets in our further analysis, but these ones will be labeled as less trustworthy data.

### Validation 1: prediction of growth and yields on single substrates

Several validation tests will be performed in subsequent sections in order to validate our *P. pastoris* FBA model*.* First, we will check if the model is able to predict growth on several substrates (glucose, glycerol and methanol). Then, we will check if the theoretical biomass yields on these substrates are in agreement with the actual yields that *P. pastoris* shows in experimental conditions.

### Simulation procedure

To predict the biomass yield we compute a set of FBA simulations, one per each substrate (glucose, glycerol, and methanol). In each simulation all substrate uptakes were fixed to be zero (thus representing the substrate unavailability) except one, which was fixed to be 1 mmol/g/h (the exact value is not important, since we will be calculating yields). Oxygen uptake was assumed to be unlimited. This way we represent a scenario where one single substrate is being consumed, no other substrates are available, and oxygen is not limited. The assumed cells objective is maximizing growth.

In summary, we are predicting how *P. pastoris* cells will be using each substrate in the selected scenarios, according to our model constraints and the assumption of growth maximization as evolutionary objective.

We performed our simulations to get the optimal flux distribution that is the model prediction (see methods). Then we compute biomass growth yields (Yx/s) based on the flux values of the optimal solution. These values are finally compared with experimental yields taken from literature. We also included the yields reported in a genome-scale model of *P. pastoris* [29]. The comparison is presented in Table 1.

**Table 1:** ***P. pastoris*** **yields in single substrates**

**Methanol**				**Glucose**			**Glycerol**		
	**Yx/s**	**Y**_**S/O2**_	**Y**_**S/CO2**_	**Yx/s**	**Y**_**S/O2**_	**Y**_**S/CO2**_	**Yx/s**	**Y**_**S/O2**_	**Y**_**S/CO2**_
	**Cmmol**	**mmol**	**mmol**	**Cmmol**	**mmol**	**mmol**	**Cmmol**	**Mmol**	**mmol**
	**mmol-1**	**mmol-1**	**mmol-1**	**mmol-1**	**mmol-1**	**mmol-1**	**mmol-1**	**mmol-1**	**mmol-1**
FBA (this work)	0.66	0.83	0.34	3.97	1.97	2.03	2.26	1.21	0.74
FBA (Caspeta)	0.49	1.43	0.49	3.91	1.53	1.96	2.23	0.95	0.68
Exp. (average)	0.42 ± 0.09	1.06 ± 0.06	0.55 ± 0.02	3.41 ± 0.66	1.44 ± 0.58	1.84 ± 0.4	1.99 ± 0.17	1.33 ± 0.27	1.01 ± 0.18

### Results

We first checked that, as expected, our FBA model is able to sustain growth on all three single substrates. Glucose, glycerol and methanol are sufficient in their own to produce all precursors and energy requirement for growth. According to the model, the best carbon source was glucose (with a yield of 3.97 Cmol dcw/mmol) followed by glycerol (2.26 Cmol dcw/mmol), and finally methanol (0.66 Cmol dcw/mmol). This ranking is in agreement with data previously reported [30], supporting the idea that the set of reactions considered in our model is capturing relatively well the main metabolic pathways *P. pastoris.*

Furthermore, the predicted biomass yields for all three substrates are found to be in reasonably good agreement with the average experimental yields of our 72 datasets, and also with the values reported for Caspeta’s genome-scale model. This provides a first validation for the model constraints and also for the hypothesis of maximal growth as cells objective, as it seems able to capture (partially, at least) the metabolic regulation that *P. pastoris* has evolved and which determines its behavior in the presence of these substrates. Notice, however, that the predicted yields tend to be larger than the experimental ones. The best agreement is shown with glycerol and glucose (around 13% overestimation), but deviation is significant with methanol (around 50% overestimation).

We suggest three tentative hypotheses to explain these last results.

Firstly, the simplicity of our model makes us disregard other operating constraints (*e.g.,* thermodynamics, availability of other nutrients, *etc.*) additional to stoichiometric and irreversibility constraints that could also influence the actual capabilities of the microorganism, resulting in actual yields lower that predicted.

Secondly, our model is not accounting for recombinant protein production, which occurs in the majority of the experiments used for validation, and which is known to affect *P. pastoris’s* use of available resources (and generally, but not always, to result in lower growth).

Finally, the assumption of growth maximization may not perfectly capture the actual cells evolutionary objectives (which may be more subtle and complex). This seems particularly likely when methanol is the substrate, since the deviation is larger in these scenarios.

All these three issues will be discussed in more depth in subsequent sections, where more data will be available.

### Validation 2: FBA predictions in real scenarios

For the next validation of our FBA model, we will define scenarios where some substrates are available (glucose, methanol, or mixes of ethanol and glycerol). Then, we will use the FBA model to predict if and how these substrates will be consumed. These scenarios correspond to our 72 datasets, so we will have data to validate the model predictions. Predictions of growth, substrate uptake, respiration rates and byproduct formation rates will be validated against experimental data in each case.

### Simulation procedure

Each scenario is defined by the availability of each substrate (glucose, glycerol and methanol), which is represented by binding their uptake to a maximum value equal to the experimental one, as reported in the corresponding dataset (v_i_ ≤ v_i,measured_). Notice that the uptake flux values are not fixed, but just bounded. To represent the unavailability of substrates their uptake flux is fixed to be zero. The oxygen uptake rate was not restricted, thus assuming that it was not the limiting factor (notice that this makes the prediction more difficult: if oxygen was indeed a limitation in some scenarios, our model will not have this information about the environment that cells are facing). As before, the objective function used in the FBA model is growth maximization.

### Results

**Figure 2:**
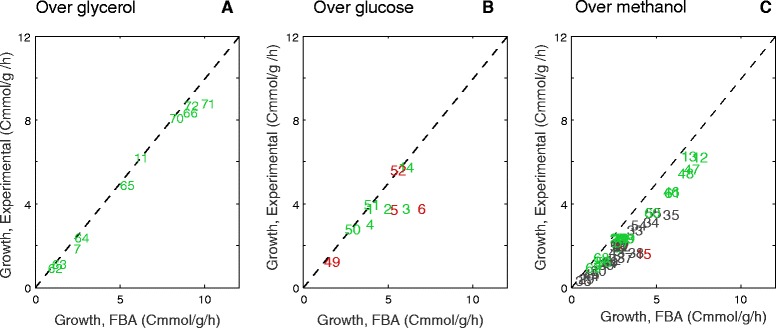
**FBA growth predictions vs. experimental growth.** Comparison of predicted growth and experimental growth for each dataset with different substrates as carbon sources: **A)** glycerol, **B)** glucose, **C)** methanol and methanol-glycerol mixtures. Green labels represent consistent datasets, whereas red ones are those classified as inaccurate. Gray represents those in which the carbon balance could not be checked.

Prediction of growth, substrate uptake, respiration rates, and byproduct formation rates are given in Figure 2 and Table 2 for each scenario. As shown in Figure 2 and Table 2, predictions of growth and substrate uptake are remarkably accurate in scenarios growing on glycerol and glucose. It seems clear that growth maximization is a quite reasonable assumption in these scenarios. It seems that substrates tend to be used through pathways that result in almost optimal growth. Notice also that byproduct formation is not predicted in any scenario, which is also in agreement with the experimental evidence.

**Table 2:** **FBA predicted fluxes vs. experimental fluxes**

**Glycerol**												
**Code**	**μ**		**Glycerol**		**Oxygen**		**CO**_**2**_		**By-products**			
	**msd** ^**1**^	**ptd** ^**2**^	**msd**	**ptd**	**msd**	**ptd**	**msd**	**ptd**	**msd**	**ptd**		
7	1.88	2.46	1.09	1.09	2.16	1.32	1.56	0.81	0.00	0.00		
11	6.17	6.21	2.75	2.75	3.62	3.33	2.35	2.04	0.00	0.00		
62	0.90	1.16	0.52	0.52	0.82	0.62	0.62	0.38	NR^3^	0.00		
63	1.11	1.39	0.62	0.62	0.87	0.75	0.65	0.46	NR	0.00		
64	2.38	2.74	1.21	1.21	1.65	1.47	1.22	0.90	NR	0.00		
65	4.89	5.42	2.40	2.40	3.12	2.91	2.29	1.78	NR	0.00		
66	8.37	9.13	4.04	4.04	4.77	4.89	3.40	3.00	NR	0.00		
70	8.07	8.31	3.68	3.68	3.99	4.45	2.96	2.73	NR	0.00		
71	8.79	10.16	4.50	4.50	4.71	5.44	4.70	3.34	NR	0.00		
72	8.66	9.17	4.06	4.06	4.19	4.91	3.53	3.01	NR	0.00		
NRMSE^4^	12%		0%		14%		23%					
Median error^5^	13%		0%		13%		24%					
**Glucose**												
**Code**	**μ**		**Glucose**		**Oxygen**		**CO**_**2**_		**By-products**			
	**msd**	**ptd**	**msd**	**ptd**	**msd**	**ptd**	**msd**	**ptd**	**msd**	**ptd**		
1	3.72	3.83	0.96	0.96	2.00	1.90	2.09	1.95	≥0.02^a^	0.00		
2	3.72	3.74	0.94	0.94	1.78	1.85	1.87	1.90	≥0.04^a^	0.00		
3	3.74	3.64	0.92	0.91	1.69	1.80	1.75	1.85	0.00	0.00		
4	3.01	3.93	0.99	0.99	2.12	1.95	2.37	2.01	≥0.03^b^	0.00		
5	3.71	5.29	1.33	1.33	1.57	2.62	2.03	2.69	≥0.3^c^	0.00		
6	3.73	6.92	1.74	1.74	0.54	3.43	1.65	3.52	≥1.0^c^	0.00		
14	5.74	6.00	1.51	1.51	2.71	2.97	3.18	3.06	0.00	0.00		
49	1.03	1.71	0.43	0.43	0.33	0.85	0.74	0.87	NR	0.00		
50	2.57	2.78	0.70	0.70	0.78	1.38	1.15	1.42	NR	0.00		
51	3.99	3.93	0.99	0.99	1.75	1.95	2.27	2.01	NR	0.00		
53	5.26	5.56	1.40	1.40	1.34	2.76	2.12	2.84	NR	0.00		
NRMSE		29%		0%		64%		32%				
% Median error		6%		0%		11%		15%				
**Methanol**												
**Code**	**μ**		**Methanol**		**Oxygen**		**CO**_**2**_		**By-products**			
	**msd**	**ptd**	**msd**	**ptd**	**msd**	**ptd**	**msd**	**ptd**	**msd**	**ptd**		
15	1.60	4.18	6.31	6.31	7.56	5.23	3.44	2.13	0.00	0.00		
28	2.32	2.66	4.02	4.02	4.22	3.33	2.33	1.36	0.00	0.00		
36	0.31	0.66	0.99	0.99		0.82		0.33	0.00	0.00		
37	1.39	3.09	4.66	4.66		3.86		1.57	0.00	0.00		
38	1.62	3.73	5.64	5.64		4.67		1.90	0.00	0.00		
39	1.04	2.00	3.02	3.02		2.50		1.02	0.00	0.00		
40	1.20	2.25	3.39	3.39		2.81		1.14	0.00	0.00		
41	1.93	2.88	4.34	4.34		3.60		1.47	0.00	0.00		
42	1.31	2.40	3.62	3.62		3.00		1.22	≥0.01^b^	0.00		
43	1.66	2.67	4.02	4.02		3.33		1.36	≥0.01^b^	0.00		
44	0.54	1.15	1.73	1.73		1.44		0.59	0.00	0.00		
45	0.66	1.11	1.67	1.67		1.38		0.56	0.00	0.00		
53	1.97	2.69	4.06	4.06		3.36		1.37	NR	0.00		
54	2.96	3.93	5.93	5.93		4.91		2.00	NR	0.00		
55	3.54	4.76	7.18	7.18		5.95		2.42	NR	0.00		
56	1.22	1.80	2.72	2.72	2.93	2.26	1.57	0.92	NR	0.00		
57	2.12	2.94	4.44	4.44	4.70	3.68	2.48	1.50	NR	0.00		
58	2.31	3.17	4.79	4.79	5.05	3.97	2.72	1.62	NR	0.00		
59	2.34	3.21	4.85	4.85	5.08	4.02	2.68	1.64	NR	0.00		
60	3.53	4.71	7.12	7.12	7.22	5.89	3.76	2.40	NR	0.00		
61	4.47	5.90	8.90	8.90	8.67	7.37	4.46	3.01	NR	0.00		
NRMSE		61%		0%		51%		45%				
% Median error		45%		0%		21%		39%				
**Glycerol methanol mixtures**												
**Code**	**μ**		**Glycerol**		**Methanol**		**Oxygen**		**CO**_**2**_		**By-products**	
	**msd**	**ptd**	**msd**	**ptd**	**msd**	**ptd**	**msd**	**ptd**	**msd**	**ptd**	**msd**	**ptd**
8	2.07	2.56	0.95	0.95	0.63	0.63	2.70	1.67	1.70	0.92	0.00	0.00
9	1.72	2.65	0.74	0.74	1.48	1.48	3.90	2.12	2.10	1.05	0.00	0.00
10	2.02	2.83	0.57	0.57	2.33	2.33	4.85	2.62	2.21	1.21	0.00	0.00
12	6.18	7.49	2.77	2.77	1.87	1.87	7.19	4.90	4.18	2.69	0.00	0.00
13	6.24	6.84	2.23	2.23	2.73	2.73	7.20	4.96	3.60	2.58	0.00	0.00
19	2.32	2.84	0.67	0.67	2.01	2.01	3.21	2.47	1.77	1.18	0.00	0.00
20	2.32	2.80	0.51	0.51	2.49	2.49	3.46	2.68	1.89	1.22	0.00	0.00
21	2.32	2.78	0.43	0.43	2.73	2.73	3.58	2.78	1.97	1.24	0.00	0.00
22	2.32	2.75	0.31	0.31	3.09	3.09	3.76	2.93	2.09	1.27	0.00	0.00
23	2.32	2.74	0.28	0.28	3.18	3.18	3.79	2.97	2.09	1.28	0.00	0.00
24	2.32	2.72	0.18	0.18	3.49	3.49	3.96	3.11	2.17	1.31	0.00	0.00
25	2.32	2.69	0.13	0.13	3.62	3.62	3.96	3.16	2.21	1.32	0.00	0.00
26	2.32	2.69	0.11	0.11	3.69	3.69	4.02	3.19	2.25	1.33	0.00	0.00
27	2.32	2.68	0.09	0.09	3.74	3.74	4.06	3.21	2.25	1.33	0.00	0.00
29	0.39	0.86	0.27	0.27	0.38	0.37		0.64		0.33	0.00	0.00
30	0.77	1.56	0.54	0.54	0.50	0.50		1.07		0.57	0.00	0.00
31	1.16	2.25	0.82	0.81	0.63	0.63		1.50		0.82	0.00	0.00
32	1.93	2.89	1.09	1.09	0.66	0.66		1.86		1.03	0.00	0.00
33	2.71	3.69	1.36	1.36	0.94	0.94		2.42		1.32	0.00	0.00
34	3.09	4.66	1.90	1.90	0.55	0.55		2.75		1.60	0.00	0.00
35	3.48	5.81	2.45	2.45	0.44	0.44		3.32		1.96	0.00	0.00
46	4.54	5.83	2.53	2.53	0.18	0.18	4.78	3.21	3.25	1.94	0.00	0.00
47	5.63	7.06	2.61	2.61	1.76	1.76	5.35	4.61	3.09	2.53	0.00	0.00
48	5.44	6.72	2.22	2.22	2.58	2.58	5.73	4.82	3.33	2.52	0.00	0.00
NRMSE		32%		0%		0%		34%		39%		
% Md error		19%		0%		0%		23%		39%		

^1^Measured values from dataset. ^2^Predicted values. ^3^Non reported values. ^4^Root mean square deviation normalized. ^5^Median of percentage errors.

Note: The datasets 1, 2, 4, 5, 6, 42 and 43 reported small quantities of byproducts. ^a^Ethanol and citrate, ^b^citrate only, ^c^ethanol, citrate and pyruvate.

Predictions of oxygen uptake rate and carbon production rate are less accurate. This may pinpoint modeling errors (in the model constraints or in the assumption of maximizing growth), but also errors in gas measurements: these measurements are generally less reliable, since they are based on determinations of the exhaust gases flow and concentration, which are prone to substantial experimental deviations.

It is also noticeable that discrepancies in methanol scenarios are larger than those in other substrates, with a median error of 45% for biomass growth (for 19% in mixes of glycerol-methanol, 12% in glycerol, and 6% in glucose). Again, this indicates that the FBA model is less precise in scenarios in which methanol is consumed. As we have already mentioned in the former section, there are several possible reasons for this behavior: (i) our underlying constraint-based model may have errors or limitation in the methanol pathways, *e.g*., reactions and other constraints may be missing, (b) our model is not considering the resources devoted to produce recombinant protein, and (c) the hypothesis of maximizing growth could be less suitable in the case of methanol, since it is a less frequent substrate in the environment for which *P. pastoris* is selectively adapted.

Let us discuss in more depth what could explain these deviations between predicted and actual cells behavior.

The first reason to explain why predicted values are larger than the measured ones is that our model is only accounting for stoichiometric and irreversibility constraints, but there could be other operating constraints such as thermodynamic constraints or biochemical restrictions resulting from regulation (*e.g.* feedback inhibition of enzymes limiting the optimal use of substrates). This applies for all three substrates; however the overestimation in methanol is larger than in glycerol and glucose, suggesting that our stoichiometric model could be not accounting for relevant skills in the methanol metabolism. For example, phenomena such as accumulation of formaldehyde and hydroxide peroxide at high methanol concentrations may result in cell growth impairment as both oxidized products of methanol are toxic for the cell [31]. Biogenesis of peroxisomes, the central metabolism organelle for assimilation and dissimilation of methanol greatly disturbs cellular content, as it can occupy 90% of the cell volume during growth in methanol [32,33]. It should also be mentioned that the biomass equation in the model was adapted from other yeast (*S. cerevisiae*) and growth conditions (glucose as the only carbon source) [28]. Exclusive growth on methanol might also represent a highly specific cellular condition that would require the development of a biomass equation of its own for an improved predictive accuracy.

However, it is still remarkable that even if our model is a raw representation of the whole metabolism and even if metabolism is only part of all phenomena occurring within cells, imposing these constraints seems to be enough to allow reasonably accurate predictions.

A second reason to explain the deviation is that the assumption of growth maximization does not perfectly represent the evolutionary objectives of these cells. This is particularly plausible in the case of methanol, because it is a less common (or frequent) substrate in nature for *P. pastoris.* If this is the case, it would be an efficient evolutionary strategy to not completely regulate every metabolic reaction if methanol is the only available substrate in a given moment, because these conditions will not remain long time, and therefore the metabolic cost of regulate and deregulate every reaction could be an inefficient effort. This reasoning is in agreement with the hypothesis that a specific flux distribution at a certain condition might be chosen to minimize adjustment efforts to other conditions, as proposed in [17]. In addition, as methanol assimilation is a highly specific capability for this yeast, not seen in most species, it could be the case that optimal growth is not required to overtake competitors in an already favorable environment.

Finally, it must be taken into account that our model is not considering recombinant protein production. This can also explain why the predicted growth tends to be larger than the observed one. Metabolic precursors and energetic resources required to produce recombinant protein, as the stress that this production provokes in cells, are not taken into account in our predictions —instead, we are implicitly assuming that recombinant strains behave as a wild type strains, and thus no heterologous protein is produced—. These phenomena penalize substrate uptake, and thus growth, and will possibly impact also growth in terms of yield (although there is evidence suggesting the opposite in scenarios where glucose is the substrate [34]). If these phenomena related with protein production were taken into account in our model, the predicted growth might be lower and show a better agreement with experimental data.

In summary, our FBA model, which couples a constraint based model with the hypotheses of maximization of growth, shows an acceptable agreement with the experimental data of dozens of chemostat cultures of *P. pastoris,* especially when glycerol and glucose are the carbon sources. Several issues must be highlighted in this regard: (1) heterogeneity within the evaluated experimental conditions (different sources, microbial strains, recombinant proteins, culture conditions), where, in addition, measurement accuracy will not always be perfect; (2) our model does not consider all constraints operating in the system, but only (partial) stoichiometry and irreversibility; (3) we are assuming that cells behavior is optimal in one particular sense —growth—, what is an extreme and rough assumption; and (4) we are not considering the effects that protein production may have on cells behavior. These factors are clearly important. Anyhow, it is remarkable that even thought this model is a crude representation of whole metabolism, and metabolism is also a limited part of all cellular phenomena, those constraints seem to be relevant enough to result in reasonably accurate predictions.

### Validation 3: predicting behavior under oxygen limitation

To continue the validation of our *P. pastoris* FBA model, we will investigate its behavior in aerobic and hypoxic conditions. First, we will check if the model is able to predict the qualitative behavior of cells for each single substrate.

### Simulation procedure

We will predict the behavior of *P. pastoris* in microaerobic and aerobic conditions for each single substrate. To study growth over glucose, the glucose uptake was limited to be less than 1 mmol/g/h, while methanol and glycerol uptakes were fixed to be zero. Then we performed a set of FBA simulations with increasing levels of available oxygen (*i.e.,* the oxygen uptake rate will be successively limited to be less or equal than 0.01, 0.02 … *etc.* up to 10 mmol/g/h). This way, a range of scenarios is represented, where glucose can be consumed, no other substrate is available, and oxygen changes from scarce, to abundant. In all these simulations the cells objective was maximizing growth. This exercise was repeated in three scenarios where only one substrate was available at a time. This way, we predict the aerobic and hypoxic behavior of *P. pastoris* over each single substrate to check if it correctly fits with actual cells behavior.

### Results

**Figure 3:**
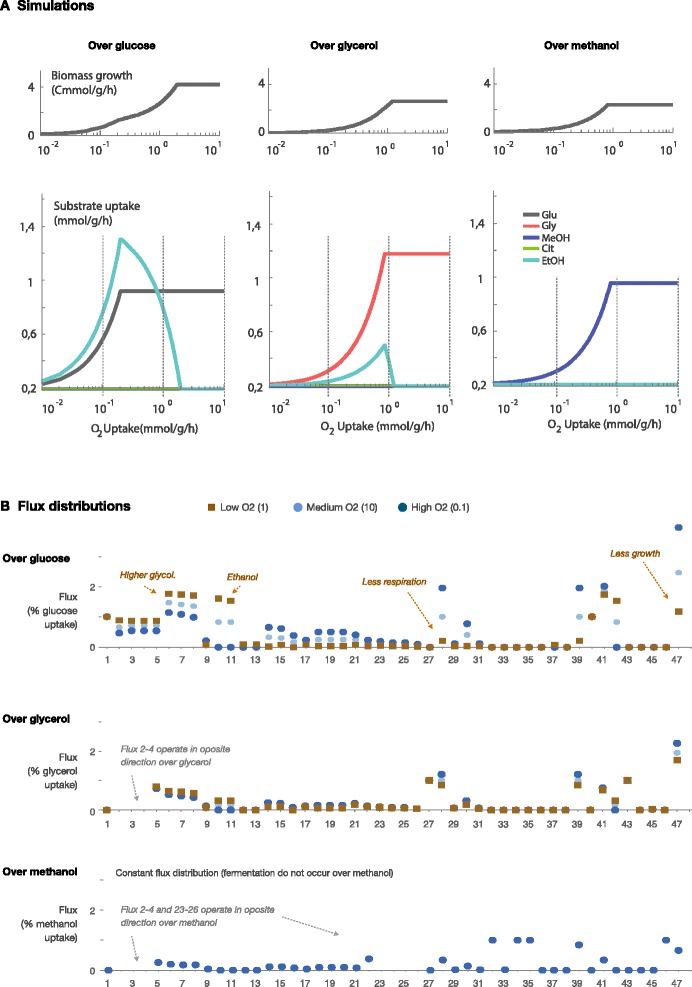
**FBA predicted behavior under oxygen limitation. A)** Biomass growth (upper panel) and substrate uptake and byproduct production (lower panel) predicted for *P. pastoris* cultures growing over a) glucose, b) glycerol, and c) methanol. **B)** Flux distributions predicted for *P. pastoris* cultures growing over glucose, glycerol, and methanol in different oxygen conditions.

The model predictions for each single substrate and different oxygen conditions are shown in Figure 3. Each graph shows the substrate uptake rate, the biomass growth rate, and byproduct production. Comparing the results, it can be observed that that glucose is predicted to be the most efficient substrate both in aerobic and microaerobic conditions (it achieves a better yield, as we already knew). Methanol will be the least efficient substrate, both in aerobic and microaerobic conditions.

Figure 3A also shows that our FBA model predicts that growth on glucose will be qualitatively different depending on oxygen availability. In microaerobic conditions, glucose is consumed via fermentative pathways (although some respiration is occurring as can be seen in Figure 3B), and thus ethanol is produced as a byproduct. These predictions are in accordance with the experimental evidence previously reported [35,36]. In those studios *P. pastoris* growth on glucose shows a facultative anaerobic behavior with oxygen limitation; however this leads to byproduct formation, especially ethanol, and also arabinitol [37]. Little information is known about the impact of oxygen availability on the physiology of recombinant yeasts, but it is well described that *P. pastoris* growth is higher in respiratory rather than fermentative mode [38]. Oxygen limitation strongly affects the core metabolism by causing energy deprivation, affecting growth, and cells have to readjust their metabolic fluxes from cellular respiration to fermentation [39].

According to our predictions, the maximum ethanol production rate will be achieved with an oxygen uptake around 0.2 mmol/g/h per 1 mmol/g/h of glucose (Y_EtOH/Glu_ = 1.53 mmol/mmol, Y_x/glu_ =1.17 Cmmol/mmol). If more oxygen is available, there is a switch from fermentative to respirative pathways —which are more efficient in terms of biomass yield, but require more oxygen—, and therefore ethanol production tends to be lower. This also makes sense from a biological standpoint. If oxygen uptake is larger than 1.96 mmol/g/h per 1 mmol/g/h of glucose, ethanol will no longer be produced, because oxygen is now in excess, and glucose can be completely consumed via respirative pathways (Y_EtOH/Glu_ = 0.00 mmol/mmol, Yx/s = 3.97 Cmmol/mmol). In this situation, the optimal growth is achieved by directing fluxes through pathways that do not involve ethanol production.

Figure 3B shows that our predictions for growth on (only) glycerol depend also on oxygen availability. The results are analogous to those obtained with glucose: ethanol is produced when oxygen is scarce, because fermentative pathways are active, but at lower rates that those predicted with glucose [40]. This agrees with the experimental evidence: even if glycerol is typically considered a non-fermentable carbon source in *P. pastoris,* residual ethanol production has been reported both in batch and fed-batch cultures [41,42]. It could be hypothesized that this lower tendency of *P. pastoris* to fermentation over glycerol with respect to glucose may be due to the extra NAD^+^ that glycerol uptake requires (in reaction 27).

Conversely, as it is shown in Figure 2C, the behavior of *P. pastoris* is different when growth is sustained on methanol: ethanol is never produced as byproduct even if oxygen is limited. Despite oxygen scarcity, our model always predicts that methanol will be consumed via respirative pathways, and never by fermentative metabolism. One obvious reason is that oxygen is required to metabolize methanol (by reaction 32), and therefore fermenting methanol is an inefficient way of getting NADH or ATP, because respiration (reaction 28) provides a better alternative—more economical in terms of oxygen— to get these resources. According to our model methanol fermentation is possible, but inefficient, and thus it is not predicted to occur.

### Validation 4: predicting substrate preferences and a behavior in hypoxic conditions

To continue the analysis of the previous section, we will now check if the model correctly predicts the preferences among multiple substrates that *P. pastoris* cells exhibit when facing an environment where oxygen is limited.

### Simulation procedure

In this simulation all three substrates were assumed to be available simultaneously. Glucose, glycerol and methanol were all limited to be less than 1 mmol/g/h. Then we performed a set of FBA simulations with increasing levels of available oxygen (*i.e.*, oxygen uptake rate was successively limited to be less or equal than 0.01, 0.02 … *etc*. up to 10 mmol/g/h). This way, we represent a range of scenarios where all substrates are available and oxygen ranges from scarce to abundant. In all these simulations the cells objective was maximizing growth. In these scenarios *P. pastoris* cells could consume the three substrates, but a preference could be shown because oxygen was limited. This way, the substrate preference of *P. pastoris* will be predicted.

### Results

**Figure 4:**
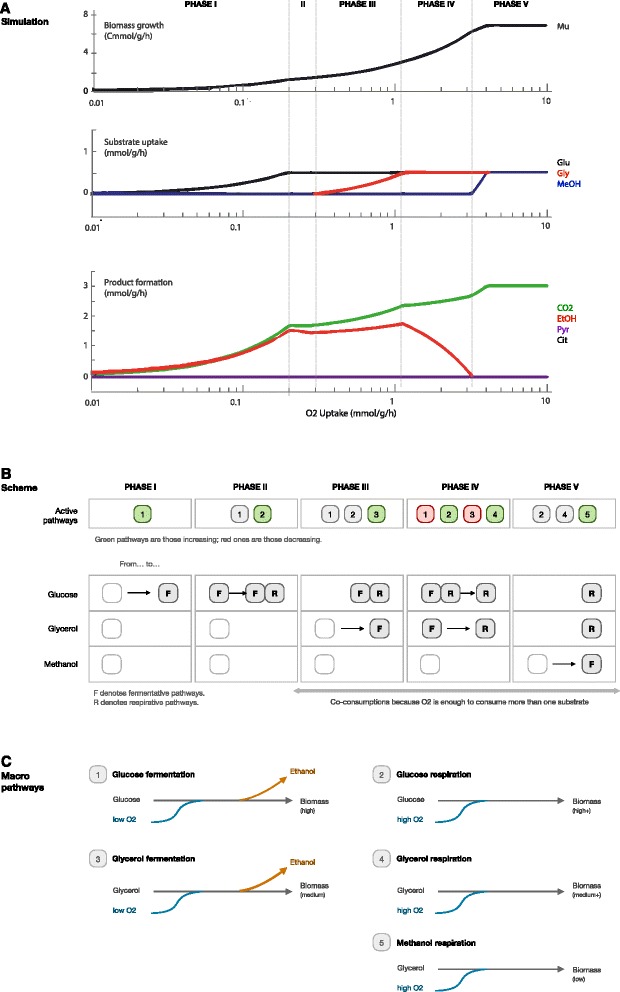
**Behavior under O2 limitation with multiple substrates. A)** Predictions of *P. pastoris* growth (up), uptakes (middle) and byproducts (down) in scenarios where the three substrates are available but oxygen ranges from scarce, to limiting condition, to abundant. **B)** Schematic active pathways in each different phase. **C)** Schematic macro pathways showed with each substrate.

The results for the battery of simulations are shown in Figure 4A. According to our FBA model, if methanol, glycerol and glucose are simultaneously fed, but oxygen is limited (less than 0.28 mmol/g/h per 1 mmol/g/h of glucose), *P. pastoris* shows a preference for glucose as carbon source. Glucose is consumed, while the others substrates are not. Simply, if oxygen availability limits the substrate uptakes, the most efficient source (in terms of yield) will be preferred. If more oxygen is available, the model predicts that glycerol will be the next substrate to be consumed, and methanol the last one. These results are in concordance with the preferences reported by Inan & Meagner —they observed that if glycerol, acetate, ethanol and methanol were present, the order of utilization was glycerol, ethanol, acetate, and finally methanol [30].

Now, let us elaborate about the four situations that our model predicts depending on how much oxygen is available. See Figure 4B and C for details about each phase.

Phase I. Cells use the first available oxygen to grow on glucose, showing a fermentative behavior that result in ethanol as by-product (pathway 1 in Figure 4B and C). This prediction is in good agreement with experimental results [35]. This behavior is shown until the oxygen is sufficient to metabolize all the available flux of glucose.

Phase II. If some more oxygen is available, glucose is still the only substrate being consumed, but now partially through respirative pathways. This implies that there is a partial metabolic switch in order to start using pathways that allow for an optimal use of glucose (in terms of growth), but that require more oxygen than those exhibited in hypoxic conditions (Phase I). As a result, the production of ethanol slightly decays. This behavior is only shown for a small range of oxygen levels: if they increase above 0.29 mmol/g/h per 1 mmol/g/h of glucose, then glycerol starts to be consumed.

Phase III. When the oxygen uptake is larger than 1.13 mmol/g/h per 1 mmol/g/h of glucose and glycerol, the FBA prediction is that glucose and glycerol will be consumed simultaneously. There is now enough oxygen to consume all the available glucose, so the “excess” is devoted to consume glycerol, while ethanol will appear as a byproduct in larger quantities —indicating that both substrates are mainly consumed through fermentative pathways (pathways 1 and 3 in Figure 4B)—.The production of ethanol and other byproducts in cultures with glycerol and glucose as carbon sources has also been reported in experimental observations [40]. The switch between phases II and III, which cannot be consequence of substrates (which do not change), could be related with NADH and ATP acting as limitants via oxygen restriction.

Phase IV. If oxygen is even more abundant, the next transition is that glycerol and glucose will be still consumed, but using the more efficient respirative pathways (the change occurs from pathways 1 and 3 to 2 and 4 in Figure 4). As a result, ethanol production tends to zero as oxygen availability increases.

Phase V. Finally, if there is more than enough oxygen to consume all the glucose and glycerol via respirative pathways, methanol is predicted to be consumed. Since methanol is the least productive substrate, the model prediction is that it will only be consumed if there are no other substrates available, or if oxygen is in high excess.

These results show that if methanol, glycerol and glucose are simultaneously fed in a limited scenario (in this case by the available oxygen), our FBA model predicts that *P. pastoris* will show a preference for glucose, followed by glycerol, and finally methanol, what is in agreement with experimental observations [41]. Notice that our FBA model is based solely on metabolic constraints and the hypothesis of maximal growth, and includes no knowledge about regulation, signaling or any other processes occurring within the cells. Remarkably, the optimality assumption is sufficient to predict (i) the substrate preference, and (ii) the use of fermentative or respiratory pathways, without representing the complex regulative machinery that cells have evolved in order to govern these processes.

Nevertheless, our FBA predictions fail in predicting co-consumptions of substrates in phases III to V. When the preferred substrate is limited (glucose) but oxygen is still available, our model predicts that the second best substrate will be consumed (glycerol). Yet, this behavior is not shown in actual batch cultures. As it is well known, when glucose, glycerol, and methanol are accumulated in culture media, they will be consumed sequentially due to enzyme regulation through catabolite repression (if the cells sense the presence of glucose, a regulation process will occur to inhibit the catabolic pathways of glycerol and methanol). The same phenomena occur when glycerol (but not glucose) is available; methanol uptake pathways will be inhibited. This catabolic regulation —which occurs at transcriptional level— is the mechanism that cells have evolved in order to implement the substrate preference that we have predicted to result in optimal growth.

But why our FBA model predicts co-consumptions when oxygen is available in excess? Or better, why cells have not evolved a machinery to show this behavior if it is predicted to be more efficient? The explanation, in our opinion, could be in our model setting, which is not accounting for other constraints limiting the “biological activity” in a broad sense, such as transport processes, enzyme production, scarcity of cellular anabolic machineries (*e.g*., ribosomes), *etc*. If oxygen or a single substrate acts as limitant, our predictions are reasonable; however, if those limits are not active at certain conditions, our model lacks the remaining constraints and tends to predict more growth (or, in general, “biological activity”) that the one actually possible. In other words, if we include in our model any kind of limiting factors, the predictions tend to be in agreement with actual cells behavior, but when these limiting factors are missing, our predictions will predict more activity than the actual one, as it happens with co-consumptions.

Finally, notice that in fed-batch cultures —where the catabolic regulation will not occur because the substrate is not accumulated and therefore cells are unable to sense its presence— *P. pastoris* cultures indeed show co-consumptions as those predicted by our FBA model. The glucose–glycerol co-consumption has been previously observed in fed-batch cultures [40], and also glycerol-methanol [41,43] and glucose-methanol [44].

Note that our objective with this last validation procedure was to get predictions from the original, raw model at different substrate environments before fine-tuning the model without considering regulation or kinetics. At this point, the limits of our simple FBA model are known, we may consider adding a minimum layer of regulation to incorporate knowledge that the model is lacking. The advantage is that now this can be done with a minimal complexity approach —that is, adding as little complexity as possible in order to further increase the model accuracy—, while keeping the optimal growth hypothesis as the main driving force of our FBA model.

## Conclusions

We have validated a small-sized FBA model of *P. pastoris* metabolism using experimental data from the literature. Our purpose was to test the model ability to give reasonable predictions in a wide range of experimental conditions without tuning the model, just applying an FBA hypothesis of maximal growth over a constraint-based model that accounts only for simple stoichiometric and reversibilities. We have intentionally avoided fine-tuning any parameter related to biomass composition, ATP assimilation, substrate preference, reaction kinetics, regulation phenomena, etc.

The computations along the paper show that our *P. pastoris* FBA model is able to (i) predict growth yields over single substrates; (b) predict growth, substrate uptake, respiration rates, and byproduct formation in scenarios with different substrates; (c) predict the behavior of *P. pastoris* in aerobic and hypoxic conditions over single substrates; and (d) predict the substrate preference under oxygen limitation.

In general, the results show that FBA model predictions based on growth maximization are reasonably accurate in many situations, particularly when glucose and glycerol are the carbon sources. The divergences with respect to the experimental data become larger in scenarios growing on methanol. We have already discussed how different causes could explain this. One possible explanation is that our model is not detailed enough. Another explanation is that our model, which represents wild-type strains, disregards the alterations that occur in modified organisms due to the production of recombinant protein. Finally, it could be that the hypothesis of maximizing growth is not as suitable growing on methanol growth as it is when cells uptake glucose or glycerol. Another limitation of our model occurs in scenarios of multiple substrates and no oxygen limitation, when it predicts co-consumptions that are not seen in actual cultures. Probably, the reason is that our model is lacking other constraints that operate in those situations. At this point, the model can be extended to improve its predictive capacity. First, methanol pathways can be detailed and the biomass equation could be revised in those conditions. Second, the expression of recombinant protein could be addressed to better represent modified organisms. Finally, we want to consider adding a layer of regulation into the model in order to better predict the cells behavior in scenarios where multiple carbon sources are available.

Nevertheless, even if (i) our FBA model is a small one, (ii) it has no parameter tuned, and (iii) it imposes a strong assumption regarding how cells regulate their metabolic fluxes (maximizing growth), it is able to provide reasonably good predictions regarding growth, substrate preference, product formation, and respiration rates in many heterogeneous experimental scenarios. In our opinion, these results suggest that small FBA models can be a valuable tool in scenarios of data scarcity —where measurable fluxes are scarce, models are small and general, and experimental data is not abundant—, which are common circumstances in industrial environments and pilot laboratories.

## Additional files

Additional file 1
***P. pastoris*** **Metabolic Network, Excel file with the list of reactions, metabolites and stoichiometric matrix.**
Additional file 2
**Experimental datasets.** Excel file with all the 72 experimental datasets taken from the literature. This file includes measurement of biomass, substrates uptakes (glycerol, glucose, and methanol), Oxygen Uptake Rate (OUR), CO_2_ production (CPR), and formation of byproducts (ethanol, citrate, and pyruvate) and Consistency analysis results [45-52].
